# Exploring a One Health approach to sustainability with international One Health and Global Health Security experts – differences, similarities and trade-offs between sectors

**DOI:** 10.1371/journal.pgph.0005225

**Published:** 2025-12-02

**Authors:** Osman Ahmed Dar, Hassaan Zahid, Max Claron, Neil Spicer, Mishal Khan

**Affiliations:** 1 Consultant in Global Health, Global Public Health Directorate, United Kingdom Health Security Agency, London, United Kingdom; 2 Medicins Sans Frontiers, Juba, South Sudan; 3 Health and Environment Programme Manager, Friendship France, Paris, France; 4 London School of Hygiene and Tropical Medicine, London, United Knigdom; 5 London School of Hygiene and Tropical Medicine, London, United Knigdom; Griffith University, AUSTRALIA

## Abstract

Sustainability in global health remains inconsistently defined and operationalised across human, animal, and environmental health sectors. As the One Health approach gains global traction—particularly in addressing complex, ‘wicked’ health problems such as pandemics, antimicrobial resistance, and ecosystem degradation—there is a growing need for shared conceptualisations of sustainability to support cross-sectoral collaboration and ultimately, long-term impacts. This study explores how One Health and health security experts from diverse disciplines understand and construct the meaning and determinants of sustainability. We conducted a qualitative study underpinned by the Social Construction Framework (SCF) through semi-structured interviews with 29 global experts from human, animal, and environmental health domains. Participants were purposively sampled from key technical advisory bodies, including the One Health High-Level Expert Panel, the World Bank Pandemic Fund, and the Quadripartite. Data were collected via online interviews between July 2023 and March 2024, transcribed verbatim, and analysed thematically using an inductive approach. Participants offered multi-dimensional definitions of sustainability; they distinguished between process-oriented (e.g., institutional longevity, financing, local ownership) and outcome-oriented (e.g., ecological regeneration, intergenerational well-being) views. Human health experts emphasised health system continuity, while animal health participants highlighted economic and disease control outcomes. Environmental experts framed sustainability around planetary resilience and equity. Cross-sectoral convergence was found on key determinants: political commitment, stable financing, workforce capacity, community ownership, and adaptability. Our findings underscore that sustainability in One Health is a socially constructed and sectorally influenced concept. Differences in framing, deservingness, and measurement priorities reflect different sectoral mandates, but also reveal potential trade-offs and synergies. To operationalise One Health effectively, an integrated sustainability framework is needed - one that aligns sectoral priorities, recognises diverse metrics, and fosters long-term, adaptive collaboration. This study provides an empirical basis for shaping such a framework, rooted in the lived experiences and perspectives of global experts.

## Introduction

The 1987 Brundtland Report, formally titled “Our Common Future,” introduced the concept of sustainable development, emphasizing the interconnectedness of economic growth, environmental protection, and social equity [1]. The report laid the foundation for sustainability science which has been described as “…an emerging field of research dealing with the interactions between natural and social systems, and with how those interactions affect the challenge of sustainability: meeting the needs of present and future generations while substantially reducing poverty and conserving the planet’s life support systems.” [2]. In the decades following the Brundtland Report, the recognition of environmental determinants of health has grown substantially. Research has increasingly focused on how factors such as air and water quality, chemical exposures, and climate change directly impact human health outcomes. Alongside this, the concept of planetary boundaries, introduced by the Stockholm Resilience Centre in 2009, has been instrumental in framing sustainability discussions. This framework identifies nine critical Earth system processes and their thresholds, beyond which humanity’s safety is compromised. Transgressing these boundaries, such as those related to climate change and biosphere integrity, poses significant risks to human health by destabilizing the ecosystems that support life [3]. Across the human health sector however, much of the literature on sustainability relates more narrowly to the processes of achieving it in relation to human health programmes, initiatives and outcomes specifically, rather than on the well-being of other species or the planet’s interconnected and inter-dependent ecosystems [4–6].

Sustainability is thus often considered a “wicked problem” due to its inherent complexity, multiple interlinked issues, and lack of a single, definitive solution. Wicked problems are characterized by complexity and interconnectedness across disciplines and sectors, unclear definitions, conflicting stakeholder interests with competing values, the need to consider trade-offs and the potential for unintended consequences [7,8]. When considering health and sustainability together these wicked problems include a broad array of global health security issues such as climate change with ecosystem damage and biodiversity loss, emerging infections including zoonoses with outbreak potential, as well as neglected tropical diseases, antimicrobial resistance, pandemic prevention and control, and food and nutrition security (including food production and supply, food safety, obesity and under-nutrition) amongst others.

To address these complex, closely connected sustainability challenges, the One Health approach -integrating human, animal, and environmental health - has over recent decades gained global recognition and become an essential framing tool [9–11]. The prominence and increasing global relevance of the approach has been clearly highlighted by the Pandemic Agreement brokered by the World Health Organization (WHO) in 2025 on behalf of its 194 Member States, where Articles 4 and 5 of the agreement focus on the One Health approach and include its endorsement in an international legally binding document for the first time [12]. The One Health approach, now well-defined (see box) and increasingly widely understood by governments and policymakers across sectors, is viewed as particularly important in low-resource settings, where fragile health systems with limited access to services, structural vulnerabilities and competing priorities, amplify both the risks and negative impacts of health-related wicked problems [9,13–16]. A strengthening evidence base has demonstrated its efficacy and cost-efficiency in more effectively controlling disease, preventing outbreaks and integrating detection, preparedness and response to a whole suite of issues. These range from the control of neglected endemic diseases such as rabies to emerging infectious diseases with pandemic potential, and other critical challenges at the human-animal-environment interface such as antimicrobial resistance, unsustainable food systems and climate change-induced natural disasters [17]. While the One Health approach now clearly dominates much of the policy and science discourse around multi-sectoral mechanisms for achieving improved health outcomes across species and as such is the focus of this paper, it is important to recognise that other related concepts have developed in parallel across the science-policy interface in recent decades, including Planetary Health and Ecohealth. While some differences exist in terms of emphasis and nuance, work has been done to bridge the conceptual and epistemic gaps between them. Centrally, the three approaches all encourage broadened perspectives on health – moving away from a purely anthropocentric outlook to one that is more eco-centric and recognises more explicitly the importance of healthy ecosystems in maintaining and improving the well-being of not just humans, but also other species and our shared environment. [9,11,17]

**The One Health Definition [11]**

*One Health is an integrated, unifying approach that aims to sustainably balance and optimize the health of people, animals and ecosystems.*

*It recognizes the health of humans, domestic and wild animals, plants, and the wider environment (including ecosystems) are closely linked and inter-dependent.*

*The approach mobilizes multiple sectors, disciplines and communities at varying levels of society to work together to foster well-being and tackle threats to health and ecosystems, while addressing the collective need for clean water, energy and air, safe and nutritious food, taking action on climate change, and contributing to sustainable development*.

With the seismic geopolitical changes to the global health and sustainable development architecture over the last year following drastic cuts in donor spending on development, the United States of America’s withdrawal from the WHO, and the broader assault on multilateral institutions, sustainability has become an increasingly important concern as programmes, institutions, services and global initiatives to support health contract and are forced to make drastic cuts in scope and reach [18]. As the world is already significantly off-track to achieving the Sustainable Development Goals (SDG) by 2030 even before the most recent funding cuts, the risks to people, animals, our ecosystems and the planet have only exacerbated further [19]. Conceptualising and developing a shared understanding of sustainability across the human, animal and environmental health sectors, is therefore, of critical importance in both operationalising One Health initiatives as well as ensuring their success and long-term positive health impacts across humans, animals and ecosystems. However, sustainability continues to be inconsistently defined, conceptualised and analysed across One Health disciplines and sectors as recent studies have shown [4,6,20–23]. At times, particularly within the human health sector, sustainability has been conceptualized to address primarily the continuation of programmes or interventions with a focus on financial and technical self-sufficiency. In other instances, there is a focus on the achievement of desired health outcomes, and in others still on the empowerment of local communities to shape their own health priorities and the related investment (see Table 1 below) [21]. By contrast, the animal and environment sectors sometimes adopt a broader, more holistic framing for sustainability considering regenerative use of resources and improved health outcomes for humans, animals and the ecosystems upon which they depend [22,23]. For several issues, including human health, animal health and food security, researchers have highlighted the need for transdisciplinary One Health approaches to assess the sustainability of interventions [10,14,15,22].

**Table 1:** Conceptual Framework of Sustainability in Health *(Adapted from Khan et al., 2019).*

Sustainability Focus	Definition	Dominant Stakeholder Perspective/ Discipline
1. Financial Self-Sufficiency and Continuity	Sustainability as the continuation of a programme or intervention using domestic resources, minimising or eliminating external support.	International aid and development; Public health programming
	Sustainability of health outcomes through improved health status, even if programmes evolve or end.	Health promotion and systems thinking
	Capacity of systems to adapt or evolve interventions based on changing circumstances.	Implementation science; Health systems research
2. Institutional and Structural Change	Embedding change through cultural shifts or structural integration of policies within institutional frameworks and routine operations.	Organisational theory; Complex systems; Health systems strengthening
3. Community Ownership and Empowerment	Programmes shaped and demanded by communities, fostering long-term behavioural change, resource mobilisation, and political accountability.	Community development; Participatory governance
4. Comprehensive/ Integrated Model	Combined model recognising financial, institutional, and community domains as synergistic levers of sustainability.	Systems thinking; Organisational resilience

Building on the current literature, this qualitative study examines the understanding and conceptualization of sustainability and its determinants as relates to the health of humans and other species, amongst an international group of One Health and health security experts from across the human, animal and environment health disciplines. The study will help efforts to develop a common understanding of sustainability across sectors, expert groups and policymakers addressing complex challenges at the human-animal-environment interface. Its findings will support sectoral integration and foster closer cooperation and collaboration amongst One Health practitioners.

## Methods

### Ethics statement

This study involved qualitative research with human participants, who were interviewed individually via online video conferencing platforms. All participants received a detailed information sheet outlining the study objectives, procedures, and their rights, including the right to withdraw at any time without consequence. Written and verbal informed consent was obtained from all participants prior to data collection. To ensure confidentiality, all data were anonymised, and no identifying information is reported. The study protocol was reviewed and approved by the Research Ethics Committee of the London School of Hygiene and Tropical Medicine (LSHTM Ethics Reference: 28748).

### Theoretical perspective

As our study sought to understand how One Health experts conceptualize sustainability, how they construct its meaning, and what they perceive as determinants within their social, institutional, and disciplinary contexts, we adopted social constructionism as our theoretical lens and the Social Construction Framework (SCF) for public policy to underpin our analysis. The SCF is premised on the belief that reality is not always objective and fixed, and that knowledge and meaning are co-constructed through social processes, language and context. Concepts such as ‘sustainability’, ‘health security’, and ‘One Health’ are not universal truths but are shaped by social, political, institutional and cultural contexts. Understanding expert perspectives therefore requires exploring how these ideas are framed, legitimized and negotiated through language, narratives, power relations between stakeholders and interactions within professional and policy communities [24–28]. With access to a global set of policymakers and subject matter experts, the study aimed to examine policy processes, social constructions and power relations when integrating and assessing the sustainability of One Health and health security initiatives across sectors, societies and geographies. This helped us understand what similarities and differences exist in its conceptualization across disciplines and how the concept of sustainability is currently being considered across international initiatives designed to help address a range of ‘wicked’ global health challenges.

### Sampling strategy and study population

We adopted a purposive sampling strategy to identify interviewees for our study. The study population consisted largely of participants from three global One Health and Health Security subject matter expert groups, namely the One Health High Level Expert Panel (OHHLEP), the World Bank Pandemic Fund (PF) Technical Advisory Panel (TAP), and the Quadripartite (World Health Organization (WHO)/World Organization for Animal Health (WOAH)/ United Nations Food and Agriculture Organization (FAO)/United Nations Environment Programme (UNEP)) One Health global technical focal points. OHHLEP is a cross-disciplinary group of international technical and policy experts drawn from every global region and competitively selected to independently advise the Quadripartite on their joint programming on One Health and other related activities across the human-animal-environment interface [29]. Similarly, The World Bank PF TAP consists of global health security experts drawn from every region of the world that independently score proposals submitted to the Fund and make recommendations to its secretariat and Board around which proposals should be supported based on technical merit and how the Fund should best operate in line with One Health principles, public health ethics and evidence-based best practice [30]. The Quadripartite are four international human, animal and environment agencies that coordinate and collaborate their activities on behalf of their Member States to pursue and advance the One Health approach at all levels. The global One Health Joint Plan of Action (2022–2026) represents the overarching strategy adopted by the Quadripartite to implement the approach at the national, regional and international levels [10]. A small number of additional subject matter experts external to these three groups were identified by participating members and also interviewed for the study.

In total, 29 participants were recruited to the study with 11 participants from a primarily human health disciplinary background, 11 others from an animal health background and 7 from an environment/ecosystem health background. In addition to human medicine, veterinary medicine and the biological sciences, areas of expertise represented by the group included; epidemiology and statistics, health emergencies and logistics, social sciences, environmental engineering and management, political science, conservation, health economics and mathematical modelling. Interviewees were drawn from every global region, including Africa, Asia, Europe, Oceania, North and South America, and categorised between those hailing from high income countries versus those from Low/Middle income countries (see Table 2). All participants had extensive experience working multi-sectorally with at least one other sector (human, animal or environment) and therefore had both experiential and subject matter expertise in other One Health related disciplines. No invited participants declined to take part in the study, however, several (N = 3) could not participate as scheduling difficulties and competing professional demands meant they were not available for interviews during the 9-month data collection window.

**Table 2:** Participants’ characteristics according to disciplinary/sector background and national identity (World Bank country classification by income level).

Sector	High Income country	Low/Middle Income Country	Count (N)
Human Health	5	6	11
Animal Health	5	6	11
Environment/Ecosystem Health	6	1	7
Total (N)	16	13	29

### Data collection and analysis

Data collection took place from July 2023 to March 2024. All participants were approached prior to the interviews by a Zoom/MS Teams call or email to obtain both verbal and written consent to participate in the study. An information sheet describing the purpose and methods of the study, and an interviewee consent form were shared at recruitment with all study participants prior to interviews. The confidentiality and anonymity of participants’ responses was highlighted and permission to be audio recorded and quoted anonymously in research outputs was requested both verbally and in writing. Participants were free to refuse any of these options, as well as withdraw from the study at any time.

Face-to-face, semi-structured in-depth interviews were conducted jointly by two of the study team trained in qualitative interviews (OAD and MC) using online video calling software through either MS Teams or Zoom. A pre-piloted interview topic guide seeking views on conceptualisations of sustainability and its determinants, as well as on similarities, differences and trade-offs between sectors and disciplines helped structure the interviews with participants (see S1 Text: Appendix A). These were kept purposely broad to avoid leading questions and to encourage interviewees to be as open as possible in sharing their personal and professional views, particularly around inter-sectoral and interdisciplinary dynamics as they might relate to elements of the SCF. In addition, the primary investigator for this study, OAD, is also a member of both OHHLEP and the PF TAP and has professional working relationships with many of the study participants. The reflexivity statement included with this study considers the way the researchers’ interactions with participants may have been influenced by their own professional backgrounds, experiences and prior assumptions (see S1 Text Appendix B).

All interviews were conducted online in English and lasted between 45–60 minutes at a timing most convenient to study participants. Audio recordings were transcribed verbatim and identifying data removed to ensure confidentiality. MS Excel was used to organize and share the data amongst study team members. As a first step we used an interpretivist approach and thematic analysis with inductive coding to identify and record emerging themes. The analysis was conducted manually and identified themes were supported by excerpts from the raw data. Coding occurred concurrently over the data collection period to enable the study team to determine when theme saturation had been reached. Three researchers (OAD, MC and HZ) independently examined the data and codes were compared and collated into potential themes and subthemes through an iterative, consensus-based process. Adopting the constant comparative method, one researcher (OAD) then conducted line by line analysis of the transcripts, naming each line and segment of data by using a separate coding frame linked to the SCF. Broad code categories included the framing of sustainability, knowledge boundaries and epistemic authority, discourse and language, perceived determinants, power and identity, tensions and contradictions, and changes over time. Adopting this approach enabled researchers to both analyse data inductively as it emerged, particularly around conceptualisations of sustainability and its determinants as well as categorise it against an SCF-based framework. Later interviews were used to test preliminary findings from earlier interviews and thematic categories further refined until all the data were analysed [31].

Our study followed consolidated criteria for reporting qualitative research (COREQ) and the checklist is included in the supplementary material accompanying this paper (see S1 Text Appendix C) [32].

## Results

Using the SCF – considering target populations, political power, issue framing, narratives, and deservingness as themes to explore – we gained key insights into how interviewees frame sustainability and its determinants. Overall, sustainability in One Health emerged as a multi-dimensional concept encompassing both the *processes* that enable long-term collaboration and capacity, and the *outcomes* of sustained health and ecological well-being. Our findings also indicated that processes and outcomes were constructed narratives that fall along a continuum that sometimes overlap at the margins, and what constitutes a process and what is distinctly an output or outcome is not always clear. Based on our study population, we present the main themes, categorised into process-related and outcome-related dimensions of sustainability. In line with a One Health approach, we also present a comparison of sector-specific perspectives including similarities, differences, and trade-offs using the dimensions of the SCF as an analytical frame.

### Conceptualisations of sustainability: process vs outcome dimensions

Most experts described sustainability in dual terms – some emphasizing *what* outcomes need to be sustained, and others focusing on *how* to sustain programs and systems. For example, KI 21 (environment health expert) stressed a triple-outcome view:

*“I view sustainability as having three dimensions: environmental, social, and economic… without sustainable practices in all three… the whole structure collapses.”*

Several interviewees from environmental and planetary health backgrounds echoed this broad framing. KI 15 (environment health expert) spoke of embedding sustainability in *outcomes and processes*, involving:

*“Three key dimensions: the environmental, the socioeconomic, and the health aspects… a sustainable system isn’t just one that maintains current conditions; it must be regenerative…natural systems must be allowed to recover and regenerate.”*

Another interviewee KI 1 (environment health expert) went further stating

*“…there’s sustainability from a life cycle perspective: the life cycle of chemicals, materials, and even pathogens and how we manage resource use and its environmental consequences.”*

On the other hand, many interviewees (especially those in public health and veterinary roles) framed sustainability in terms of process – the continuity of programs, capacities, and collaborations. For instance, KI 22 (human health expert) described viewing sustainability in short-, medium-, and long-term phases, where the goal is to embed capacities into domestic systems so that:

*“Even when international or donor funds recede, the systems remain in place.”*

KI 16 (animal health expert) similarly defined sustainability as a long-term commitment supported by:

*“Reliable, long-term funding and…sustainable structures… institutional frameworks… that remain robust over time.”*

From a health security angle, KI 8 (human health expert) citing the HIV/AIDS response argued: *“successful responses were underpinned by political support and sustained funding; without these, even effective interventions can collapse. Additionally, workforce development and leadership are essential…expertise is retained over decades.”*

Notably, participants recognised both sides of this coin. Several explicitly remarked that experts define sustainability in varied ways – some prioritising outputs/outcomes (e.g., health indicators, environmental quality) while others stress the durability of the intervention itself. As one interviewee KI 23 (human health expert) observed:

*“Some look at outputs and outcomes… while others see it as a process, questioning whether an intervention is sustainable.”*

Overall, the consensus was that sustainability in One Health must integrate both dimensions: the long-term maintenance of desired health, environmental and social outcomes, and the continuation and adaptation to as necessary, of the locally appropriate and culturally tailored structures, resources and partnerships needed to achieve those outcomes.

### Key process-related determinants of sustainability

A number of process factors were identified by interviewees as critical to ensuring One Health initiatives endure and adapt over time and are outlined below.

#### Stable long-term financing.

Perhaps the most frequently cited factor was sustained funding. Interviewees stressed moving beyond short project cycles and donor-driven bursts. KI 28 (environment health expert) when commenting on developing environmental laboratory capacity in LMIC settings opined:

*“One of the biggest challenges is ensuring that these capabilities are sustained after our direct involvement and once our funding diminishes. Funding is crucial—not only for acquiring and maintaining expensive equipment and reagents but also for the infrastructure, such as data storage and service contracts, necessary to keep these systems running.”*

Another linked sustainability directly to the funding timeframe of projects:

*“Often, projects run for three to six years and then funding ends abruptly, leaving initiatives unsustained,…sustainable funding is essential”* said KI 16 (animal health expert).

Integrating programs into domestic budgets was viewed as vital

*“When donor resources end, it’s essential that these activities are absorbed into the national budget… so that their positions are maintained over time.”* KI 22 (human health expert)

#### Political will and governance.

Strong political support, leadership, and institutional buy-in were highlighted as enabling factors critical for sustainability, alongside technical and financial factors. Several participants advocated for formal institutional frameworks and policies to lock in multisectoral collaboration. For example, KI 11 (human health expert) argued that:

*“Strong legal framework, backed by formal agreements… is crucial to maintain sustainability and resilience over time.”*

#### Workforce and capacity building.

Many interviewees saw investment in human resources and technical capacity as fundamental. This includes training and retaining skilled staff across sectors and developing the next generation of One Health practitioners. KI 11 (human health expert) linked sustainability to resilience in terms of workforce:

*“having skilled staff… and strong collaboration with academia and research underpins sustainability.”*

Continuity of expertise through succession planning was also highlighted so that expertise:

*“is retained over decades.”* KI 8 (human health expert)

Furthermore, workforce sustainability was viewed as needing to be developed hand-in-hand with other capacity building investments as KI 1 (environment health expert) pointed out:

*“There’s also workforce sustainability—the retention of skilled staff and the importance of continuous training—which, unfortunately, is rarely incorporated into investments. I’ve seen examples where solar panels work well in countries with established pipelines, whereas in others, rapid infrastructure build-up leads to installation without proper maintenance.”*

#### Cross-sectoral partnerships and collaboration.

Interviewees stressed breaking down silos and institutionalising channels for information sharing and joint action. For instance, KI 11 (human health expert) recounted a One Health success in Pakistan where rapid sharing of information about wildlife deaths led to discovering a toxic water source affecting humans – made possible by a platform that convened agriculture, veterinary and public health experts. The lesson was that:

*“a country-level platform…regular and ad hoc meetings among stakeholders is critical for implementing a sustainable One Health approach.”*

KI 12 (animal health expert) noted that in global One Health projects, ensuring each sector sees value is key:

*“we ensure we address the specific needs of each partner… so that no sector feels sidelined. If an intervention only benefits one sector, it’s unlikely to be sustainable.”*

#### Integration into existing systems, appropriateness and local ownership.

Several experts argued that sustainability requires embedding One Health into national systems and fostering local ownership. Sustainability was often linked to how well One Health activities become part of the routine fabric of institutions. In practice, this might mean mainstreaming One Health in ministry budgets, standard operating procedures, or curricula.

KI 18 (human health expert) added that sustainability demands community co-ownership:

*“it has to be ‘owned’ by the people who are affected by it; the beneficiaries must also be the implementers.”*

Several mentioned exit strategies for projects that transfer responsibilities to local authorities without leaving gaps. Critically one participant KI 2 (human health expert) noted:

*“Appropriateness means that an intervention must fit within the local cultural and sociocultural milieu. It’s not just about the technical aspect; it’s about ensuring that the approach resonates with local norms, values, and practices.”*

Along similar lines KI 28 (environment health expert) recollected:

*“If local partners and stakeholders care about the tools and the work, they will drive progress and help sustain the initiative. We’ve seen success in countries that secure local funding and have a dedicated workforce that “runs with it” even after our support tapers off.”*

#### Adaptability and evolution.

A subtle but important determinant was the ability to adapt over time. Interviewees noted that sustainability does not imply doing the exact same thing forever; rather, programs should evolve as contexts change. *“Sustainability doesn’t mean continue forever… maintain the system until it naturally evolves into something new or is no longer necessary in its original form.”* KI23 (human health expert)

This calls for flexibility and learning within initiatives. For example, KI 25 (animal health expert) highlighted the need for systems that are:

*“self-generating and resilient over time.”*

This was seen as akin to natural ecosystems, rather than rigid high-input systems that eventually degrade. Several also alluded to fostering a sustainability mindset culturally, so that organisations and communities value and continue the efforts.

### Outcome-related dimensions of sustainability

Interviewees commonly stressed that One Health should ultimately produce lasting improvements in health and environment – not just short-term wins. Several outcome dimensions were emphasised and are elaborated on below.

#### Holistic health and well-being outcomes.

Many experts lamented that too often one domain’s outcomes dominate.

*“In practice…language of threat and security tends to dominate…resulting in narrow, securitized interventions that favour detection and rapid response over…holistic wellbeing across all sectors.”* said KI 13 (human health expert)

A sustainable outcome would instead be equitable and long-term well-being for all species. Several interviewees invoked the One Health High-Level Expert Panel definition [9]. A number of participants explicitly argued for evaluating sustainability in comprehensive terms: beyond biological conservation of species, including social well-being and economic viability. Some even within human health also urged a shift toward preventive, health-promoting outcomes (e.g., healthier lifestyles and environments) rather than just low disease incidence.

#### Intergenerational and long-term impact.

Another key outcome dimension was temporal – sustainability is inherently about the long term. Many participants rooted their definition in protecting the future.

*“I see it as a practical approach to ensure a future where my grandchildren and their descendants can have a future,”* said KI 25 (animal health expert)

Some participants framed it as a “resilience” outcome: the planet remaining liveable for future generations, not just maintaining current project outputs. This future-oriented view translates to outcomes like resilient health systems and ecosystems that can absorb shocks over decades. KI 14 (environment health expert) argued that truly sustainable outcomes sometimes require present-day sacrifice:

*“if we truly aim for long-term sustainability, we must accept some discomfort or sacrifice...”*

Many also pointed to environmental regeneration as a yardstick of success – e.g., recovery of wildlife populations, cleaner air and water maintained over time – rather than just short-term risk reduction. As one example, KI 15 (environment health expert) highlighted the need for:

*“a broader cultural and systemic shift toward regeneration and balance.”*, shifting from wasteful practices to circular, regenerative ones.

#### Multi-domain metrics and balance.

A theme related to outcomes was how they are measured and balanced. Several interviewees noted each sector tends to prioritise its own metrics of success. *“Human health is driven by immediate service delivery… animal health by production and infectious disease control… environmental health by ecological boundaries and regenerative capacities.”* (KI 15 Environment health).

If pursued in silos, each might achieve its targets but collectively undermine sustainability. One Health sustainability requires composite indicators or shared goals that reflect co-benefits. For instance, food security interventions should be assessed not only on tons of food produced or human nutrition improved, but also on environmental impact and animal welfare. Ultimately, interviewees advocated for balancing metrics and seeking “win–win–win” (across people, profit, planet) scenarios where possible. KI 14 (environment health expert) described sustainability as:

*“…a mathematical optimization problem.”*

Narratives of deservingness were also apparent here: interviewees from all sectors acknowledged that historically human health outcomes are treated as most “deserving” of attention, often at the expense of animals or ecosystems. A sustainable One Health vision, however, challenges that hierarchy by asserting the inherent value (and necessity) of animal welfare and ecosystem integrity for long-term health. The collective view was that sustainability means recalibrating this balance – expanding the circle of target beneficiaries to genuinely include animals and environment, not only as means to human ends but as worthy of protection in their own right, in policy and practice.

### Comparing perspectives by sector: human, animal, and environment health

#### Human health:

Interviewees rooted in human health tended to focus on sustaining the health system functions and outcomes for human populations. They often framed sustainability in terms of strengthening health services, surveillance, and preparedness in a way that can be maintained. Several stressed integrating One Health activities into national health systems and budgets, highlighting the need to preserve gains in disease control beyond emergency phases. This reflects the health sector’s orientation toward measurable targets and continuous service delivery.

A common thread in human health perspectives was an anthropocentric tilt – a candid recognition that their primary mandate is to save human lives and improve human well-being. This is reinforced by training and institutional mandates. Human health interviewees often justified One Health in terms of protecting human health from threats: zoonoses, food safety issues, or environmental risks. This is linked to the issue framing in global health security – e.g., zoonotic disease control is framed as necessary to prevent human pandemics, climate action is framed by health co-benefits. KI 13 (human health expert) commented on this framing, noting that when One Health is put into practice:

*“...the language of threat and security tends to dominate…favouring detection and rapid response”* (to human outbreaks) *rather than preventive work on environment or long-term wellness.”*

Despite this human-centric focus, human health professionals in the interviews strongly supported the One Health approach and recognised their sector cannot achieve sustained outcomes alone. Many underscored the interdependencies with examples from practice. KI 22 (human health expert) speaking of rabies control warned of focusing only on human vaccination or post-bite treatment:

*“without addressing animal health, you may miss a critical prevention opportunity, whereas vaccinating dogs yields long-term human and animal benefit*.”

Human health interviewees also highlighted that their sector often enjoys relatively greater political power and funding compared to veterinary or environmental agencies, placing a responsibility on them to bring others along.

*“Human health is often the best funded and most prioritized… by contrast, animal health is framed in economic terms and environmental health is often marginalized until a crisis.”* KI 13 (human health expert)

The challenge in their view was to use the leverage of the health sector to ensure sustained attention (and resources) for the others. In practice, human health experts spoke of working closely with agriculture and environment colleagues so that One Health initiatives produce co-benefits. Several noted in multisectoral projects they always consider sector-specific outcomes (animal or environmental) alongside public health goals, because an intervention only seen to benefit humans will not get buy-in or last.

Our analysis found the human health perspective on sustainability is grounded in maintaining and scaling health programs that protect people, with an emphasis on funding, governance and efficiency. They largely acknowledge the trade-off that if they focus exclusively on immediate human outcomes, they might undermine longer-term sustainability – hence the need to invest in prevention at the animal/environment source and to ensure other sectors’ concerns (like livelihoods or wildlife conservation) are addressed. The human health interviewees often acted as pragmatists: they want One Health to deliver results for human populations but realise this will not happen unless the whole system (animal and environment sectors included) is made resilient.

#### Animal health:

Experts from the animal health sector offered a perspective centred on disease control in animals, livestock productivity, and zoonotic risk management, often tied to economic considerations. Many noted that sustainability in animal health is frequently viewed through an economic or utilitarian lens. Thus, sustaining veterinary interventions often means proving their value in terms of healthier herds, stable food supply, or trade benefits.

A notable difference in perspective is that animal health experts sometimes struggle for political attention unless a disease threatens humans or food security directly. Several interviewees from this sector implied their work is under-resourced unless it aligns with economic or human health interests. Animal health agencies may not have the same budget lines for “sustainability” or long-term capacity that public health does, unless an external driver (like an epidemic affecting humans or a major livestock economic crisis) prompts it. KI 23 (human health expert) noted that during outbreak responses:

*“the animal sector sometimes struggles to secure financial backing unless there is a significant economic impact, as we saw with Rift Valley fever.”*

Wildlife health and conservation-oriented interviewees (a subset of animal health sector) added yet another nuance. Some animal health interviewees emphasised ecological balance and species health as part of the equation, effectively aligning closely with environmental perspectives. KI 25 (animal health expert) warned of the unsustainability of high-input agricultural systems and argued for more regenerative practices:

*“many human systems are unsustainable because they require constant, high-cost inputs…a sustainable system is one that can renew itself with minimal intervention.…livestock production…often becomes unsustainable when pushed to the limits of its biological resilience.”*

He advocated looking at animal production systems critically in terms of long-term viability and impact on ecosystems. This shows that within the animal health sector, those focused on livestock might emphasise productivity and food security, whereas those focused on wildlife and conservation emphasise ecosystem health and the risks of overexploiting animals. However, both subgroups share concern for animal populations as targets of One Health and raise the point that without sustaining animal health and habitat, human gains are fragile.

In terms of power and deservingness, animal health experts often felt their sector was a “middle child” – not as politically powerful as human health, yet more directly tied to human interests than environmental sector. They frequently justified sustainability needs by pointing to co-benefits for humans (e.g., controlling brucellosis in livestock to protect farm workers and milk supply) or for economies (healthy livestock for livelihoods). The narrative is that target populations in animal health (e.g., farmers, livestock) are deserving of support not only in their own right but because they underpin human well-being. As one trade-off example, one interviewee described the COVID-era struggle with SARS-CoV-2 in mink farms: no single agency wanted responsibility, yet it affected public health and trade. The resolution involved balancing farmer economic interests, animal welfare (avoid mass culling unless truly needed), and public health risk.

Animal health interviewees called for more consistent support (not just crisis-driven) and integration with human health efforts so that gains (like elimination of a zoonotic disease in animals) are mutually reinforcing.

#### Environment health:

Those interviewees affiliated with environmental health, ecology, or biodiversity brought perhaps the broadest and most holistic perspective on sustainability. Consistently, they stressed that sustainability is not merely a programmatic concern but a planetary imperative. Several articulated a three-pronged view (environment, society, economy) and argued that ignoring any one prong undermines overall outcomes. Environmental experts were keen to change the perception of the environment as just a source of risks. Many in this group emphasized regeneration, balance, and natural resilience. One key difference in the environmental perspective was how deservingness and value are assigned: there was strong implicit advocacy that ecosystems and wildlife have intrinsic value and must be sustained, not only because they serve humans. This often came through via narratives and examples. KI 14 (environment health expert) gave a striking example of a cascade effect: a veterinary pharmaceutical that protected livestock led to the death of 90% of vultures in India, causing ecological and cultural damage – a consequence of valuing cattle health and farmer income over an undesirable scavenger species, until the damage was done. KI 21 (environment health expert) similarly challenged a purely anthropocentric approach by questioning if eradicating all infectious disease (a typical public health outcome) is always desirable, since:

*“infectious diseases are a natural part of biodiversity. Should we aim to eliminate them entirely, or is that counterproductive? There are always trade-offs.”*

This reflects the ecological understanding that some level of disease in ecosystems may be natural and even necessary for species population control, highlighting a philosophical divergence from the human health goal of disease eradication at all costs.

Environmental health interviewees also discussed issue framing differences. A few noted that even within environmental circles, the concept of “environmental health” can be too narrowly construed as pollution and toxicology, missing the broader ecosystem picture.

*“Environmental experts may have a very narrow view of ‘environmental health’ as toxicology or pollution metrics…rather than a broader ecosystem or biodiversity perspective,”* observed KI 15 (environment health expert), based on experience in global forums.

KI 16 (animal health expert) echoed this, saying:

*“environmental health still struggles with a narrow view… mainly considering toxicology or pollution metrics, rather than holistic assessment of ecosystem resilience or biodiversity”.*

This demonstrated a diversity of views within the environment sector, where some focus on environmental exposures affecting human health (e.g., air and water quality – often under health security mandates), and others (like conservationists) focus on wildlife and ecosystems themselves. The interviewees in our sample generally leaned toward the ecosystem/planetary health side, advocating that sustainability metrics should include ecological boundaries and regenerative capacity. They encourage reframing One Health not just as a way to prevent environmental risks to people, but as a way to protect the environment for its own sake and for mutual benefit. As one noted:

*“the environment has historically been on the receiving end…often seen as an externality, and One Health is an opportunity to change that by “minimizing environmental damage—or even creating positive outcomes through cross-sector work.”* KI 21 (environment health expert)

Environment sector interviewees often highlighted trade-offs and win-wins in terms of resource use, consumption patterns, and policy choices. They frequently brought up examples involving climate, food systems, and pollution.

In terms of power dynamics, environmental professionals often find themselves least empowered in One Health discussions, which historically were driven by disease and health security agendas. This was acknowledged in interviews: environment tends to be heard when there isa crisis (e.g., climate disasters, pandemic origins being environmental) but otherwise struggles for funding. As KI 13 (human health expert) quipped:

*“Environmental health is often marginalized until a crisis occurs.”*

The narrative of deservingness here is that ecosystems rarely have a direct voice or political constituency, so part of sustaining One Health is giving the environment a seat at the table permanently, not just when “needed” instrumentally. The environmental health experts in our study pushed for a proactive approach: including environmental indicators in monitoring, securing dedicated funding for environmental components of One Health, and culturally valuing prevention (like maintaining ecosystem services) as much as response.

### Trade-offs and synergies in a One Health Approach

All groups – human, animal, and environmental – acknowledged that pursuing an integrated One Health approach involves navigating trade-offs as well as identifying co-benefits. A consistent theme was that unbalanced approaches lead to trade-offs that undermine sustainability, whereas a One Health approach seeks to recognise and manage these trade-offs transparently. The common trade-offs identified by our expert group of interviewees are described below:

#### Short-term vs Long-term.

Focusing on short-term results (e.g., emergency disease containment) can draw resources away from building long-term capacity or prevention.

*“If you invest heavily in a short-term project – say, emergency response – you achieve rapid results but fail to build lasting structures for long-term sustainability…focusing exclusively on reducing disease incidence might come at the expense of broader ecosystem health.”* KI 16 (animal health expert)

Many human health experts echoed this, recognizing the pattern of surge funding that disappears post-crisis. The trade-off is immediate impact vs sustained resilience. The unanimous recommendation was to leverage short-term wins to strengthen the system continuously rather than simply reacting in an ad hoc manner, thereby turning a potential trade-off into a synergy (using outbreaks as opportunities to reinforce systems).

#### Human benefits vs Environmental costs.

Perhaps the most frequently illustrated trade-off was between maximizing a human health or economic benefit and causing collateral environmental damage. KI 22 (human health expert) discussed a vivid example asking rhetorically if we should *“triple-wrap frozen chicken to prevent one additional case of Campylobacter… even if it means a huge amount of plastic waste?”*, highlighting a minor human health gain at a major environmental cost. Similarly, KI 10 (environment health expert) and others spoke of *“agricultural intensification”* or industrial policies that improve human outcomes (food, income) but degrade ecosystems – a trade-off unsustainable in the long-term. The general agreement was that better cross-sectoral dialogue can highlight these trade-offs early and inspire innovative solutions that minimize harm to any sector – true “co-benefits” approaches.

#### Human vs Animal health priorities.

Within One Health, there can be tension between human and animal health actions. Rabies control was used as an example by multiple interviewees: investing in mass dog vaccination (animal health effort) versus investing in human post-exposure prophylaxis – how to balance budgets between the two? The sustainable solution, everyone agreed, is to upstream the effort (vaccinate dogs *and* educate communities) to eventually reduce human cases, even if in the short run human health sector might prefer funding only human vaccines. Many noted that one sector acting alone often did not even see the trade-off – it becomes apparent only when sectors plan together.

#### Local vs Global priorities.

KI 22 (human health expert) pointed out a different kind of trade-off:

*“the difference in how issues are managed at various levels. Local-level responses might be more agile and tailored, whereas national or international responses can be more bureaucratic.”* Limited resources force prioritisation at each level, and sometimes global agendas do not align with local community needs. A sustainable approach would try to reconcile these – ensuring global initiatives are flexible enough to adapt and be appropriate locally, and local successes inform broader policy. This trade-off highlights that scale matters in One Health – sustainability requires alignment from community through to global governance, otherwise efforts might stall when moving from pilot to policy.

#### Integration vs Specialisation.

An insightful trade-off mentioned by KI 8 (human health expert) is between pushing cross-sector integration versus maintaining specialised excellence. *“If we push for broad, cross-sectoral strategies too early, we risk diluting the technical expertise critical in each field,”* he said.

This suggests a balance: we need integrated planning, but also depth of knowledge. The solution proposed by several is interdisciplinary teams where each sector brings its best, rather than merging roles too much. In other words, human doctors, vets, and ecologists should collaborate (so integration at the problem-solving level) but still do what they each do best technically. Sustaining One Health means sustaining the capacity within each sector *and* the connections between sectors.

### Offsetting trade-offs

The One Health approach inherently seeks synergies to offset these trade-offs. Many interviewees provided hopeful narratives of win-win scenarios. Several remarked on the importance of “health arguments” to promote climate action – framing climate interventions as health-enhancing, creates synergy between environmental and health sectors.

*“When we recognize how environmental factors impact all forms of health, that’s a way to advance policies that bridge gaps between sectors”* said KI 27 (environment health expert)

This kind of issue framing could turn a potential trade-off (economic development vs environment) into a mutual gain (green development for health). All sectors agreed that a better understanding and appreciation of interconnectedness enables a move from trade-offs to balance and co-benefits. None suggested that one sector’s goals should trump others permanently. Even the staunchest human health advocates in the sample acknowledged that, for example, mass culling of animals or heavy environmental pollution is a pyrrhic victory for human health. Similarly, environmental advocates acknowledged that draconian environmental policies could harm human livelihoods if not carefully implemented. KI 21 (environment health expert) for example commented that:

*“creating protected areas might displace local communities, affecting their livelihoods, even though it benefits wildlife conservation.”*

Similarly, KI 21 (environment health expert) reflecting on the history of conservation and the assertiveness of the environment sector in both the colonial and early post-colonial period recalled:

*“Nearly every protected area in Africa involved the displacement of people. In some cases, entire tribes were displaced or even lost due to the establishment of these areas.”*

For participants the trade-off discourse thus reinforced why a One Health, multisectoral approach is needed – it provides a platform to discuss and consciously balance these competing demands, rather than each sector pursuing its agenda in isolation. As KI 15 (environment health expert) stated:

*“when sectors work in isolation, they focus on optimizing outcomes in one domain at the expense of others… only by acknowledging the trade-offs and creating systems that balance these can we achieve sustainable outcomes for all.”*

## Discussion

Our study reveals a nuanced understanding of “sustainability” across disciplines and perspectives that spans maintaining programmes and processes with achieving broader long-term outcomes. In comparing expert insights with the literature, sustainability is a multi-dimensional concept across the human, animal and environment health sectors. Our findings suggest that understanding sustainability in a One Health context may require an integrated framework – one that combines the process-oriented capacities needed to carry on collaborative work across sectors with the outcome-oriented vision of a healthier planet. Table 3 below represents such a framework based on the theoretical underpinning and findings from our analysis.

**Table 3:** A One Health integrated framework for sustainability.

Framework Component	Description	SCF Lens	Sectoral Relevance/ Notes
**Shared Values**	Core principles: regeneration, resilience, equity, co-benefits, ethical stewardship across species and time	Narratives, Deservingness	Cross-cutting across all sectors; promotes mutual respect and systems thinking
**Process Enablers**			
- Stable Financing	Long-term, cross-sectoral funding aligned with national systems	Power, Target Populations	Human health often dominates funding; animal/environment sectors need sustained allocations
- Political Commitment & Governance	Legal frameworks, institutional backing, and policy integration	Political Power, Framing	Human health holds more influence; need to elevate animal/environment roles
- Institutionalisation & Workforce	Embedding One Health into systems and investing in skilled, intersectoral capacity	Target Populations, Narratives	Important for long-term resilience; succession planning key
- Cross-Sector Collaboration	Platforms for regular coordination, shared goals, and information exchange	Issue Framing, Power	Facilitates mutual understanding and reduces duplication
- Local Ownership & Appropriateness	Cultural fit, community co-creation, and domestic leadership in OH efforts	Deservingness, Target Populations	Enhances relevance and continuity; cited by all sectors as a sustainability necessity
- Adaptability & Learning	Flexibility to evolve as contexts change; promotes regenerative systems	Narratives, Framing	Embraced especially by environmental health as a model for systemic renewal
**Outcome Goals**			
- Holistic Well-being	Inclusive health outcomes for humans, animals, and ecosystems	Narratives, Deservingness	Counters siloed metrics; requires cross-sector indicators
- Intergenerational Equity	Sustaining systems and environments for future generations	Narratives, Framing	Especially emphasised by environmental health experts
- Balanced Metrics	Co-benefits and trade-offs considered using composite indicators	Issue Framing	Examples: integrating animal welfare in food production indicators
- Intrinsic Value of Nature	Recognising non-instrumental worth of ecosystems and wildlife	Deservingness	A call to shift away from human-centred valuation in One Health
**Trade-offs & synergies**			
- Short-term vs Long-term	Rapid response vs systems building	Framing	Reconcile via surge-to-system recovery transitions
- Human vs Environmental Gains	Risk of environmental harm for marginal human benefits (e.g., excessive plastic, industrial farming)	Deservingness	Balance via co-benefit innovations
- Human vs Animal Health	Budget allocation: downstream treatment (humans) vs upstream prevention (animal vaccination)	Target Populations	Rabies vaccination example used frequently
- Local vs Global Agendas	Misaligned policies; top-down programmes ignore local context	Power, Framing	Polycentric governance and feedback loops recommended
- Integration vs Specialisation	Risk of losing technical depth if integration is overextended	Framing, Narratives	Advocated for interdisciplinary teams with retained sectoral expertise

### Conceptions across human, animal, and environmental health

**Diverse emphases by sector:** Human health experts in our study often framed sustainability in terms of programmatic continuity and capacity. This view aligns with much of the public health literature that defines sustainability as the continued delivery of interventions and maintenance of health benefits over time. In contrast, those from an environmental background spoke of sustainability in the sense of ecological balance and intergenerational equity echoing the Brundtland Commission’s seminal definition of sustainable development. Animal health experts tended to emphasise economic and livelihood aspects, seeing sustainability in terms of resilient food systems and veterinary services that safeguard both animal and human wellbeing. Despite these different emphases, the interviews showed a strong convergence around a number of themes including a long-term view, stakeholder ownership, and adaptability. A unifying dimension was the importance of breaking out of silos – experts repeatedly stressed that sustainable outcomes in one sector depend on cooperation with the others. This aligns with the One Health approach itself, which “aims to sustainably balance and optimise the health of people, animals and ecosystems” as well as Elinor Ostrom’s insights on collective action: effective management of common resources (be it a community forest or a One Health programme) requires inclusive governance, trust, and co-production of solutions by stakeholders rather than simply top-down control [33]. Ostrom’s principles – such as stakeholder engagement, locally adapted rules, and cross-level linkages – were implicit in many interviews where experts spoke of community engagement and local “ownership” as keys to sustaining One Health initiatives.

**Cross-Sectoral convergence:** Both interviewees and literature agree that certain enabling factors are universally critical for sustainability, regardless of sector. Key among these are:

#### Political commitment, legal frameworks and policy support.

This mirrors Schell et al.’s (2013) framework, which identified *political support* as a core domain of a programme’s capacity to sustain [34].

#### Stable financing.

The need for secure, long-term funding was perhaps the most frequently cited determinant of sustainability in the interviews and is a key factor in sustainability literature [35,36].

#### Partnerships and community ownership.

One Health experts viewed broad stakeholder engagement – from government ministries to local communities – as the glue that keeps initiatives relevant and supported. This aligns with Ostrom’s idea that engaging resource users in decision-making yields better long-term compliance and outcomes and reflects findings from Wiltsey-Stirman et al. that leadership engagement, champions for the issue, and stakeholder involvement are recurrent predictors of an innovation’s continuation [37].

#### Organisational and workforce capacity.

Many interviewees underlined the importance of institutionalising skills and infrastructure and respect for diverse expertise. Building such capacity ensures that when project funding cycles or personnel change, the capability remains. This corresponds to several of Schell’s sustainability capacity domains (e.g., *organisational capacity*, *communications*, *strategic planning*) and what Lennox et al. (2020) describe as the internal factors that healthcare organisations must cultivate to sustain interventions (such as workforce development, leadership, and a culture of learning) [34,35].

#### Adaptability and learning.

The ability to adapt interventions over time was another recurring theme. This echoes the Dynamic Sustainability Framework and the notion of operating “at the edge of chaos” – i.e., successful sustainable systems find a balance between stability and flexibility [38,39]. Literature increasingly recognises that *adaptation* is not a threat to sustainability but a prerequisite for it. In practice, this means One Health initiatives should not be static: as ecological and epidemiological circumstances change and knowledge advances, so must priorities, interventions and programmes.

### Divergence in perspectives and framing

While many determinants are shared, there are some notable divergences in how sectors tend to conceptualise sustainability along process-oriented and outcome-oriented views:

#### Process-Oriented (Means).

In public health contexts, sustainability is often treated as a *process or capacity*. The emphasis is on whether the intervention or collaboration can keep going. Frameworks like Schell’s and tools like the Program Sustainability Assessment Test focus on measuring an organisation’s capacity to sustain its activities (through the domains noted above) [34,40]. Wiltsey Stirman et al. (2012) similarly looked at presence of policies, training, leadership, fit with organisation, etc., as indicators that an innovation will be sustained [37]. In these models, success = continuation: if a health task force or surveillance system is still functioning years later, that is a win.

#### Outcome-Oriented (Ends).

In environmental health and global sustainability discourse, sustainability is more often framed around *desired outcomes* – essentially the state of the world we want to achieve or maintain (e.g., biodiversity preserved, climate stabilized, health security achieved). From this perspective, it is not enough that a programme continues; what matters is whether it continues to deliver the intended benefits and not cause harm elsewhere. The planetary health concept championed by Rockström et al. exemplifies this: it sets quantifiable planetary boundaries (for climate change, biodiversity loss, nitrogen cycles, etc.) that define a “safe operating space for humanity” [3]. The implication is that human activities (including health initiatives) must stay within these outcome limits to be truly sustainable. The Lancet One Health and Global Health Security series picks up a similar thread, arguing that One Health must ultimately protect health in humans, animals and ecosystems in tandem, rather than narrowly aiming to keep a project alive [41]. In practice, outcome-oriented approaches often push for metrics and indicators to track progress towards sustainability goals – for example, Pakzad & Osmond (2016) developed multi-dimensional indicators (ecological, health, socio-cultural, etc.) to assess sustainability of urban infrastructure projects [42]. In agriculture, H. El-Bilali (2018) emphasises the need for transitions in agro-food systems towards more sustainable outcomes like improved food security, reduced environmental impact and social wellbeing [43]. These perspectives ask what are we sustaining and why?

This process–outcome divergence was evident in the expert dialogues. Some interviewees, especially those with field project experience, talked about sustaining the means – e.g., keeping labs open, retaining staff, securing funding streams. Others zoomed out to the ends, discussing sustainability in terms of impact and ethical obligations – e.g., ensuring communities are healthier and ecosystems more resilient over many years. One interviewee KI 15 (environment health expert) expertly merged the two, saying sustainability “*becomes a measure of resilience and an ethical obligation to ensure long-term well-being,”* achieved through building systems that can be maintained and handing over ownership responsibly.

#### Sectoral Priorities – Trade-offs.

Divergent perspectives also arise from the differing immediate priorities of each sector. Human health interventions often prioritise urgent outcomes (saving lives, treating patients now) and can treat sustainability as secondary (to be dealt with via handover once the emergency passes). Environmental initiatives, conversely, inherently prioritise the long view and may criticise the short-termism of the health sector. Bridging this gap requires reframing sustainability as a common goal: for example, a One Health approach to pandemic prevention is increasingly touted as a way to achieve immediate health security and longer-term environmental protection (by tackling drivers like deforestation and wildlife trade). Perry et al. (2018) explicitly argue this in the context of livestock: improving animal health through vaccines, disease control and better veterinary governance contributes to sustainable food systems by increasing efficiency (economic and food security outcomes), reducing the environmental footprint of livestock (environmental outcome), and improving farmer livelihoods (social outcome) [22]. However, realising these synergies requires overcoming process challenges – getting typically separate sectors to plan and act together, share resources, and perhaps forego a short-term gain for broader benefits. Here again Ostrom’s collective action principles are relevant: building trust and respect, aligning incentives, and ensuring no sector feels it is “losing out” by contributing to another’s goals. [33].

### Toward an integrated sustainability framework

Our primary research study is the first of its kind to analyse conceptualisations around sustainability and its determinants with a group globally influential policy and subject matter experts from across the human, animal and environment health sectors. Its findings should inform and strengthen the evidence base for future initiatives at testing and developing integrated multi-sectoral assessments of sustainability. There is growing recognition that siloed approaches to sustainability (either purely institutional or sectoral) are insufficient. Instead, hybrid models are emerging. In implementation science, frameworks are evolving to consider *context* and *adaptation* as central. These acknowledge that sustaining an intervention is not an isolated technical task, but an ongoing process influenced by its environment [5,6,30,31], while in sustainability science and policy, there is momentum towards concepts like “regenerative sustainability.” [23]. This advocates going beyond maintaining the status quo to actively restoring and regenerating systems. Applied to One Health, a regenerative approach would not just aim to keep a project running, but to leave lasting positive legacies – for instance, rejuvenating an ecosystem service while also strengthening public health infrastructure. This aligns with what some experts describe as building back better and innovating for greater future capacity, shifting from surviving to thriving [3].

Bringing these threads together, we see that points of convergence across sectors and frameworks include the importance of system thinking, equity (temporal equity for future generations in Brundtland’s sense, and cross-sectoral and cross-societal equity now), and resilience/adaptability. Points of divergence – such as differing primary objectives or time scales – can be reframed as complementary strengths if managed well. The literature suggests practical ways to do this: for example, using sustainability indicators that cover health, economic, and ecological dimensions can help teams jointly track progress, satisfying each sector’s need for accountability [42]. Adopting governance models that allow for polycentric decision-making can empower local actors in human, animal, and environmental communities to take collective ownership of problems, building trust and buy-in that sustain efforts beyond external project timelines [33]. And importantly, narrative and language matter – as one expert noted, we need to transform our language so that “sustainability” is not just a donor buzzword about funding, but a shared ethical commitment across disciplines and cultures. The challenge and opportunity for One Health is to develop a common understanding around sustainability and merge this into a unified framework where durable processes lead to durable outcomes. Further qualitative research work with One Health experts, practitioners and communities of practice as well as systematic reviews of the sustainability literature within and across sectors will be needed to fully validate such a framework. The convergence of ideas across human, animal, and environmental health shows a path forward: collaborative, systems-based, and future-oriented. At the same time, acknowledging divergences is crucial; for One Health to truly sustain, it must balance the urgency of health security with the patience of ecosystem stewardship and cultivate the “sustainability mindset” across all sectors.

## Supporting information

S1 Text Appendix A: Semi-structured interview topic guide. Appendix B: Reflexivity statement. Appendix C: Consolidated criteria for reporting qualitative studies (COREQ); 32-item checklist.
(DOCX)
