# Supplementary material for: Exploring a One Health approach to sustainability with international One Health and Global Health Security experts – differences, similarities and trade-offs between sectors
Source: PLOS Glob Public Health. 2025 Dec 2;5(12):e0005225. doi: 10.1371/journal.pgph.0005225 (PMC12671812; doi:10.1371/journal.pgph.0005225)
Supplement: S1 Text — Appendix A: Semi-structured interview topic guide. Appendix B: Reflexivity statement. Appendix C: Consolidated criteria for reporting qualitative studies (COREQ); 32-item checklist. (DOCX) [file pgph.0005225.s001.docx]

**Appendix A in S1 Text: Semi-structured interview topic guide**

**Study title: Exploring A One Health approach to sustainability – differences, similarities and trade-offs between sectors**

Thank you for agreeing to take part in this interview. My name is ________ and I am a researcher from the London School of Hygiene and Tropical Medicine (LSHTM). I am part of a team are carrying out a study to develop an analytical framework for assessing the sustainability of One Health initiatives. The first part of the interview focuses on how sustainability is conceptualised across human, animal and environmental sectors. The second part of the interview examines how the concept of sustainability is applied in a health security context by exploring its use within two widely used national capacity assessment tools – the World Health Organization Joint External Evaluation (JEE) and the World Organization for Animal Health Performance of Veterinary Services (PVS). The documents summarising mentions of ‘sustainability’ in the two tools were shared with you through email already. I hope you have had a chance to look through these to help frame our discussion.

This interview will be audio-recorded and will take approximately 50-60 minutes.

Interviewee name:

Interviewee gender:

Interviewee nationality:

Interviewee geographical/country of origin:

Interviewee institutional affiliation/job title:

Interview start and end time:

**READ THROUGH THE INFORMED CONSENT FORM AND TAKE WRITTEN CONSENT BEFORE PROCEEDING.**

Part 1

1. Could you tell me a bit about your job and area of expertise?

*Probe if needed:* What sector does your professional role most closely identify with - Human, Animal or Environmental health? Does your role involve work across more than one sector?

2. What is your understanding of the One Health approach?

3. I would like to understand your views on the concept of ‘sustainability’ as it relates to One Health. For your discipline, what are the key characteristics and determinants of sustainability in the context of multisectoral One Health initiatives?

4. How does this compare to other One Health sectors (i.e. from human, animal, environmental health) in your view? What is important to them?

*Probe if needed:* What similarities or differences do you see in perspective between your own sector and those others?

5. What do you see as important trade-offs when considering health holistically between humans, animals and the environment?

**Appendix B in S1 Text: Reflexivity statement**

This study was conducted by a research team comprising individuals with professional and academic backgrounds in global health, humanitarian and health emergency response, social sciences, and One Health. The primary investigator, who led the design, data collection, and analysis phases, has extensive experience in human health, One Health and global health in emergency settings. This background shaped the study’s focus on the sustainability of One Health approaches and framed the inquiry through a health systems and operational policy lens. The findings were shaped not only by participants’ responses, but also the researchers’ epistemological stances, disciplinary orientations, and engagement with the broader literature on health systems, environmental health, and policy discourse.

Importantly, the primary investigator (OAD) held professional relationships with many of the 29 expert participants, which facilitated a high degree of rapport and trust. This familiarity created an environment in which interviewees appeared more comfortable expressing their views candidly. While this ‘insider status’ within these high-level professional groups may have facilitated access to key informants and fostered trust during interviews this positionality may also have introduced subtle biases in framing questions, interpreting responses, implicit shared assumptions and navigating power dynamics with participants who share similar professional milieus. The research team attempted to mitigate these biases through several strategies: iterative piloting of the interview guide, reflexive memo writing, peer debriefing, and transparent coding procedures that allowed for reflexive questioning of our interpretations.

The multidisciplinary composition of the research team offered both strengths and limitations. Several members brought extensive experience in global health and humanitarian response, and others had disciplinary foundations in One Health, health systems and health policy research. The combinations allowed for a nuanced appreciation of sectoral silos and cross-disciplinary tensions, which are central to the study of sustainability in a One Health context. At the same time, the team’s relative grounding primarily in the human health sector may have influenced the framing of interview questions, the thematic priorities identified during analysis, and the interpretation of concepts articulated by participants from the animal health and environmental sectors.

Of the 29 interviewees, 11 were from human health, 11 from animal health, and 7 from environmental or ecosystem health. While this distribution ensured disciplinary breadth, the greater familiarity of the research team with human health systems may have affected how comfortably and confidently perspectives from the animal and environment sectors were interpreted, potentially underemphasizing aspects unique to food production, animal well-being, ecological sustainability or biodiversity governance.

Throughout the research process, the team adopted a constructivist stance, recognizing that concepts of sustainability are not fixed but are socially constructed through professional norms, institutional mandates, and disciplinary logics. The team approached the research with an underlying assumption that sustainability within One Health cannot be meaningfully understood without accounting for power asymmetries, institutional dynamics, and intersectoral coordination. This background likely sensitized the analysis to certain themes—such as equity, funding longevity, co-benefits, socioecological equilibrium between species and policy coherence across sectors —while potentially obscuring others that fall outside multisectoral paradigms.

In acknowledging these positional dynamics, we aim to enhance transparency and support readers in contextualizing the study findings. We recognize that all knowledge production, particularly in qualitative research involving expert informants, is shaped by the positional relationships between researchers and participants. This reflexivity statement therefore serves as a critical lens through which the study’s insights on sustainability in One Health should be interpreted.

**Appendix C in S1 Text: Consolidated criteria for reporting qualitative studies (COREQ): 32-item checklist**

| **No. Item** | **Guide questions/description** | **Reported on Page #** |
| --- | --- | --- |
| **Domain 1: Research team and reﬂexivity** |  |  |
| *Personal Characteristics* |  |  |
| 1. Inter viewer/facilitator | Which author/s conducted the interview or focus group? | 7-10 |
| 2. Credentials | What were the researcher’s credentials? E.g. PhD, MD | 7-9 |
| 3. Occupation | What was their occupation at the time of the study? | N/A |
| 4. Gender | Was the researcher male or female? | N/A |
| 5. Experience and training | What experience or training did the researcher have? | 7-9 |
| *Relationship with participants* |  |  |
| 6. Relationship established | Was a relationship established prior to study commencement? | 7-9 |
| 7. Participant knowledge of the interviewer | What did the participants know about the researcher? e.g. personal goals, reasons for doing the research | 7-9 |
| 8. Interviewer characteristics | What characteristics were reported about the inter viewer/facilitator? e.g. Bias, assumptions, reasons and interests in the research topic | 7-9 |

| **Domain 2: study design** |  |  |
| --- | --- | --- |
| *Theoretical framework* |  |  |
| 9. Methodological orientation and Theory | What methodological orientation was stated to underpin the study? e.g. grounded theory, discourse analysis, ethnography, phenomenology, content analysis | 7-9 |
| *,Participant selection* |  |  |
| 10. Sampling | How were participants selected? e.g. purposive, convenience, consecutive, snowball | 7-9 |
| 11. Method of approach | How were participants approached? e.g. face-to-face, telephone, mail, email | 7-9 |
| 12. Sample size | How many participants were in the study? | 7-9 |
| 13. Non-participation | How many people refused to participate or dropped out? Reasons? | 7-9 |
| *Setting* |  |  |
| 14. Setting of data collection | Where was the data collected? e.g. home, clinic, workplace | 7-9 |
| 15. Presence of non-participants | Was anyone else present besides the participants and researchers? | N/A |
| 16. Description of sample | What are the important characteristics of the sample? e.g. demographic data, date | 7-9 |
| *Data collection* |  |  |
| 17. Interview guide | Were questions, prompts, guides provided by the authors? Was it pilot tested? | 7-9 |
| 18. Repeat interviews | Were repeat inter views carried out? If yes, how many? | N/A |
| 19. Audio/visual recording | Did the research use audio or visual recording to collect the data? | 7-9 |
| 20. Field notes | Were ﬁeld notes made during and/or after the interview or focus group? | 7-9 |
| 21. Duration | What was the duration of the inter views or focus group? | 7-9 |
| 22. Data saturation | Was data saturation discussed? | 7-9 |
| 23. Transcripts returned | Were transcripts returned to participants for comment and/or correction? | N/A |
| **Domain 3: analysis and ﬁndings** |  |  |
| *Data analysis* |  |  |
| 24. Number of data coders | How many data coders coded the data? | 9,10 |
| 25. Description of the coding tree | Did authors provide a description of the coding tree? | 9,10 |
| 26. Derivation of themes | Were themes identiﬁed in advance or derived from the data? | 9,10 |
| 27. Software | What software, if applicable, was used to manage the data? | 9,10 |
| 28. Participant checking | Did participants provide feedback on the ﬁndings? | N/A |
| *Reporting* |  |  |
| 29. Quotations presented | Were participant quotations presented to illustrate the themes/ﬁndings? Was each quotation identiﬁed? e.g. participant number | 11-23 |
| 30. Data and ﬁndings consistent | Was there consistency between the data presented and the ﬁndings? | 11-23 |
| 31. Clarity of major themes | Were major themes clearly presented in the ﬁndings? | 11-23 |
| 32. Clarity of minor themes | Is there a description of diverse cases or discussion of minor themes? | 11-29 |
